# Impact of Operational Factors, Inoculum Origin, and Feedstock Preservation on the Biochemical Methane Potential

**DOI:** 10.3390/bioengineering8110176

**Published:** 2021-11-05

**Authors:** Audrey Lallement, Aline Siaud, Christine Peyrelasse, Prasad Kaparaju, Blandine Schraauwers, Samuel Maunas, Florian Monlau

**Affiliations:** 1 APESA, Pôle Valorisation, Cap Ecologia, Avenue Fréderic Joliot Curie, 64230 Lescar, France; audrey.lallement@apesa.fr (A.L.); aline.siaud@gmail.com (A.S.); christine.peyrelasse@apesa.fr (C.P.); blandine.schraauwers@apesa.fr (B.S.); samuel.maunas@apesa.fr (S.M.); 2 School of Engineering and Built Environment, Nathan Campus, Griffith University, Brisbane, QLD 4111, Australia; p.kaparaju@griffith.edu.au

**Keywords:** anaerobic digestion, inoculum origin, feedstock conservation, kinetic rate

## Abstract

Anaerobic digestion for the valorization of organic wastes into biogas is gaining worldwide interest. Nonetheless, the sizing of the biogas plant units require knowledge of the quantity of feedstock, and their associated methane potentials, estimated widely by Biochemical Methane Potential (BMP) tests. Discrepancies exist among laboratories due to variability of protocols adopted and operational factors used. The aim of this study is to verify the influence of some operational factors (e.g., analysis frequency, trace elements and vitamins solution addition and flushing gas), feedstock conservation and the source of inoculum on BMP. Among the operational parameters tested on cellulose degradation, only the type of gas used for flushing headspace of BMP assays had shown a significant influence on methane yields from cellulose. Methane yields of 344 ± 6 NL CH_4_ kg^−1^ VS and 321 ± 10 NL CH_4_ kg^−1^ VS obtained from assays flushed with pure N_2_ and N_2_/CO_2_ (60/40 *v*/*v*). The origin of inoculum (fed in co-digestion) only significantly affected the methane yields for straw, 253 ± 3 and 333 ± 3 NL CH_4_ kg^−1^ VS. Finally, freezing/thawing cycle effect depended of the substrate (tested on biowaste, manure, straw and WWTP sludge) with a possible effect of water content substrate.

## 1. Introduction

Anaerobic digestion (AD) is considered to be a sustainable waste management technology to produce biogas from organic wastes such as municipal, industrial, agricultural, and agro-industrial wastes. With an aim to achieve circular economy and sustainable development, the interest in application of AD technology in wastewater and organic waste management industry has gained interest in the last few decades. To assess the methane yield of a given feedstock and to design the industrial biogas plant, it is important to determine the methane potential of the different organic wastes. Biochemical Methane Potential tests (BMP) have been used as the most popular protocol employed to estimate the maximum methane production of organic substrates and to design future installations [1]. In addition to maximum methane production data, BMP tests also give information about the kinetics of methane production [2]. As BMP test is a relatively simple batch assay, it is used extensively by both the scientific community and in the industrial sector. Besides several inter-laboratory campaigns at the National and European level, no specific norms were defined for this test but some specific recommendations have been drawn [3,4,5,6,7]. There are several factors that may influence the anaerobic biodegradability of organic materials, and some of them are at present, only poor understand.

Indeed, BMP testing can be affected by a set of factors that can be classified as: set-up of the assay, environment in the assay, monitoring, data quality, and reporting [1,8,9,10]. One of the most studied parameters regarding the BMP protocol is the establishment of the inoculum to substrate ratio (ISR). The choice of ISR is generally based on the substrate composition and an ISR of 2 is applied for nonspecific substrates, 4 for easy ones and 1 for recalcitrant substrates [3,5]. With respect to the BMP assay preparation, an inter-laboratory study has highlighted the importance of the use of basic anaerobic nutrient medium to ensure BMP reproducibility [3]. However, addition of basic anaerobic medium containing trace elements and vitamins is highly dependent on the inoculum characteristics and/or source [9]. Another parameter of significance in BMP assay preparation is the type of gas used to flush the headspace in order to create anaerobic condition in the assays. Flushing with an inert gas (without oxygen) is crucial to create anaerobic condition in the assay. The gas used can be pure nitrogen [6] or a mixture of nitrogen (N_2_) and carbon dioxide (CO_2_), with 20% [8,11] to 40% CO_2_ [12]. Although use of CO_2_ in the flushing gas mixture has been shown to increase the methane production [13,14], no such results were noticed in inter-laboratory studies [15,16]. In parallel, analysis frequency can show other parameters that can affect final methane production. To evaluate the methane production, two measurements are essential: first, volume to estimate the biogas production (by water displacement or manometric measurement) and then gas composition to estimate the methane production. Gas chromatography is quite usual for biogas analysis but variation can be linked to temperature, water vapor content, and methane/carbon dioxide lose during venting [9,17]. However, little information is available in the literature regarding the frequency of this analysis, some publication analyzed gas composition for each manometric measurement [17] and other publications do not specify the frequency.

The quality of inoculum used in AD is dependent on its origin (from wet or dry anaerobic digestion process), process operating conditions, temperature (mesophilic or thermophilic), and on the type of feedstock and its composition (agricultural waste, industrial waste, sewage sludge or household waste). The start-up can be faster without an initial lag phase if the inoculum is degassed [13] and contains high microbial diversity. Inoculum with high microbial diversity can quickly adapt to new substrates [18,19,20]. Therefore, previous reactor operation parameters are important (e.g., feedstock used, process temperature, last time of feeding) and can influence the kinetics and methane production from the new substrate [9,21,22]. Previously, inter laboratory studies have tried different strategies to estimate the impact of inoculum source on methane production [4,15,23]. In an European inter-laboratory study no clear effect of inoculum origin were observed in the 19 laboratories that used their own inoculum [15]. From another international inter-laboratory study, two inocula were used: one supplied by the host lab and the other one the usual inoculum used in the respective laboratory. No evidence was found between the two sets of inoculum explaining the difference in methane production obtained [4]. Furthermore, in inter-laboratory study, the impact of laboratory (protocols, technician, and methods) can affect the result and their comparison.

Substrate conversion is also known to influence the methane production [24]. Use of fresh substrate is considered to be the best option although it is not always possible due to logistics [5,6,25]. For a short-term storage, storage of substrate at 4 °C is an ideal solution. For a long-term storage, freezing/thawing of the substrate has shown to be best option in order to prevent deterioration of the organic matter by micro-organisms [12]. Nevertheless, freezing process has shown to cause changes in the physical state of water and the formation of ice crystal can damage and/or modify the substrate structure [26]. However, this effect depends on the substrate and its initial water content. Freezing/thawing has shown to have no effect on the biogas production from green waste [12,27] and a decrease in biogas production with maize (−17%) and straw (−9%) [25]. On the contrary, an increase in biogas production by 38% was noticed with WWTP sludge [28] and 42% with food waste [26].

The lack of standards and the numerous factors influencing the BMP can limit the comparison of the results between different laboratories. In this paper, the effect of BMP protocol factors (i.e., flushing gases, analysis frequency, trace elements and vitamin addition) on the methane yields of cellulose at 38 °C was investigated. Later, the effect of inoculum source and substrate storage on the methane potential of different organic wastes (i.e., bio-waste, cow manure, straw and WWTP sludge) was evaluated.

## 2. Materials and Methods

### 2.1. Inoculum and Substrate

In this work, two types of inocula were used, both run in co-digestion process. The first inoculum (Inoculum 1) was cultivated in APESA laboratory (Lescar, France). The reactor was fed with green grass and WWTP sludge and operated under mesophilic conditions. The second inoculum (Inoculum 2) was collected from a mesophilic digester fed with slaughterhouse pig slurry, silage and industrial wastes. Inoculum characteristics are given in Table 1 (before degassing).

Both inocula were subjected to degassing by incubating the inocula at 38 °C without feeding for 4 and 6 days for inoculum 1 and 2, respectively. Degassing will limit the endogenous biogas production from the inoculum (blank). The characteristics of inoculum 1 were similar to those recommended by the European Inter-laboratories study [4]. However, it was not the case with inoculum 2. Indeed, Hafner et al. (2020) have recommended that the inoculum should have a vs. content of 15–40 g·L^−1^, pH of 7.0–8.5, total volatile fatty acids of <1.0 g·L^−1^ (as acetic acid), total ammonia-nitrogen concentration of <2.5 g·L^−1^, and alkalinity of >3 g·L^−1^ (as CaCO_3_).

Cellulose (Tembec^®^), cow manure (local farm), sewage sludge (Véolia WWTP, Lons, France; after centrifugation but collected before flocculation), wheat straw (Biofib’jardin, Sainte-Gemme-la-Plaine, France) and biowastes (e.g., blood, meat, maize, and bean from XL ADOUR METHA, Aire-sur-l’Adour, France) were used as substrates. The characteristics and theoretical methane potential of the substrates are presented in Table 2.

Regarding freezing/thawing cycle, feedstock are frozen at −15 °C during 5 days then they are thawed at 6 °C for 3 more days before BMP analysis. 

### 2.2. Biochemical Methane Potential Protocol

The BMP protocol used in this study was adapted from the European inter laboratory studies, in which APESA was involved [3,4,5]. BMP tests were performed in 500 mL glass bottles (Duran group, Mainz, Germany) with 300 mL working volume. To each assay, substrate, inoculum and water were added to achieve an inoculum to substrate (ISR) ratio of 3.9 ± 0.001. The mixture contained at least 2 g of VS of substrate. To create anaerobic conditions, headspace in the assays was flushed with pure nitrogen (99.9% N_2_) for 30 seconds at a flow rate of 2.5 L min^−1^. This flow rate corresponds to 5 times the headspace volume. Prepared assays were incubated statically at 38 °C. All substrates were tested in triplicate. Assays with inoculum alone was used as blank control. Methane produced from the blank was subtracted from the sample assays. The experiment was terminated when the daily gas production in the assays was lower than 0.5% of the total production for 3 consecutive days.

Biogas production was monitored three times per week using a manometer (Digitron 2023P, Digital Instrumentation Ltd., London, UK). Before each sampling, assays were manually shaken. Without specific indications, the gas composition was analyzed once a week by gas chromatography (Varian GC-CP4900 Agilent, Germany) fitted with a thermal conductivity detector and equipped with two columns. A Molsieve 5A PLOT column operated at 110 °C in order to separate O_2_, N_2_ and CH_4_ and a HayeSep A column operated at 70 °C and separated CO_2_ from the other gases. Injector and detector temperatures were 110 °C and 55 °C, respectively. The calibration was carried out with two standard gas composed of 9.5% CO_2_, 0.5% O_2_, 81% N_2_ and 10% CH_4_ and 35% CO_2_, 5% O_2_, 20% N_2_ and 40% CH_4_ (special gas from Air Liquide^®^, Paris, France). An extrapolation of methane concentration is done within the week. All results are presented in normalized conditions of temperature and pressure (0 °C and 1 atm).

### 2.3. Details of the Operational Modification of the BMP Protocol

The effect of operational factors such as trace elements and nutrients supplementation, type of flushing gas used and frequency of gas analysis on the methane potential tests was evaluated. Trace elements and vitamins solution were provided by the Inter-laboratory study assay (IIS-BMP project [4]) and were proposed by [8]. Briefly, the trace element solution consists (in g L^−1^) of FeCl_2_ (2), H_3_BO_3_ (0.05), CuCl_2_ (0.038), MnCl_2_ (0.05), (NH_4_)_6_Mo_7_O_24_ (0.05), AlCl_3_ (0.05), CoCl_2_ (0.05), NiCl_2_ (0.092), EDTA (0.5) and Na_2_SeO_3_ (0.1). Vitamin solution was composed of 2 mg L^−1^ of biotin, 2 mg L^−1^ of folic acid, 10 mg L^−1^ of pyridoxine acid, 5 mg L^−1^ of ridoflavin, 5 mg L^−1^ of thiamine hydrochloride, 0.1 mg L^−1^ of cyanocobalamine, 5 mg L^−1^ of nicotinic acid, 5 mg L^−1^ of P-aminobenzoic acid, 5 mg L^−1^ of lipoic acid and DL-pantothenic acid. The solutions were added to obtain a final medium of 0.1%.

Two different types of flushing gases were tested: pure N_2_ and a mixture of 40% CO_2_–60% N_2_. All gases were provided by Air Liquide^®^. The head space volume was renewed five times at a flow rate of 2.5 L min^−1^ for 30 s. For assays with inoculum 1, assays with cellulose (positive control) and assays without substrate (blank control) were also incubated. Finally, the gas analysis composition was also analyzed with two modalities: gas analysis one time per week or each day.

### 2.4. Modeling and Statistical Analysis

Kinetics of biodegradation, hydrolysis constant, and methane yields were determined using a first order kinetic model calculated according to the following equation:

V_CH4_ (t) = Vmax (1 − e^−kt^) (1)

where V_CH4_ (t) is the volume of methane produced at time t (d), expressed in NL CH_4_ kg^−1^ VS, Vmax was the maximum producible methane volume (NL CH_4_ kg^−1^ VS) and k was the methane generation rate constant (d^−1^).

Theoretical BMP were calculated for all substrates using the elemental composition (CcHhOxNnSs) [29].

(2) $$Y_{CH4}=\frac{22.4\;\left(\frac{c}{2}+\frac{h}{8}-\frac{x}{4}-\frac{3n}{8}-\frac{s}{4}\;\right)}{12c+h+16x+14n+32s}$$

where *Y*_CH__4_ is the maximal methane yield (L CH_4_ kg^−1^ VS) and 22.4 is the molar volume of an ideal gas at STP conditions (L STP mol^−1^).

Regarding statistical analysis, Student tests are performed on PAST software (ver. 2.17c; downloaded in April 2021). 

### 2.5. Analyses

Inoculum were characterized for pH, redox, TS and VS content, alkalinity, and concentrations of ammonia and volatile fatty acid (VFAs) and substrates for TS and VS content. pH was monitored using a pH meter 340i fitted with a Sentix^®^ electrodes (WTW, Weilheim, Germany). Redox was measured using a WTW 1970i apparatus (WTW, Weilheim, Germany) coupled with a platinum electrode (SI analytics^TM^, Xylem Analytics, Mainz, Germany). Total and volatile solids were determined as per the APHA Standard Method [30]. Alkalinity was determined by titration with 0.1 M sulfuric acid using the protocol described elsewhere [31]. The concentration of ammonia in the samples was determined as per the instructions of NTK kit of WTW by spectrophotometry (Photolab S6, WTW, Weilheim, Germany). Finally, VFA were determined using a gas chromatography system (7980B Agilent, Steinheim, Germany) fitted with a flame ionization detector (FID) detector and a DB–FFAP column (Agilent, Steinheim, Germany) following a temperature gradient from 90 °C to 240 °C.

## 3. Results and Discussion

### 3.1. Influence of the Operational Factors on BMP Results

**Figure 1:**
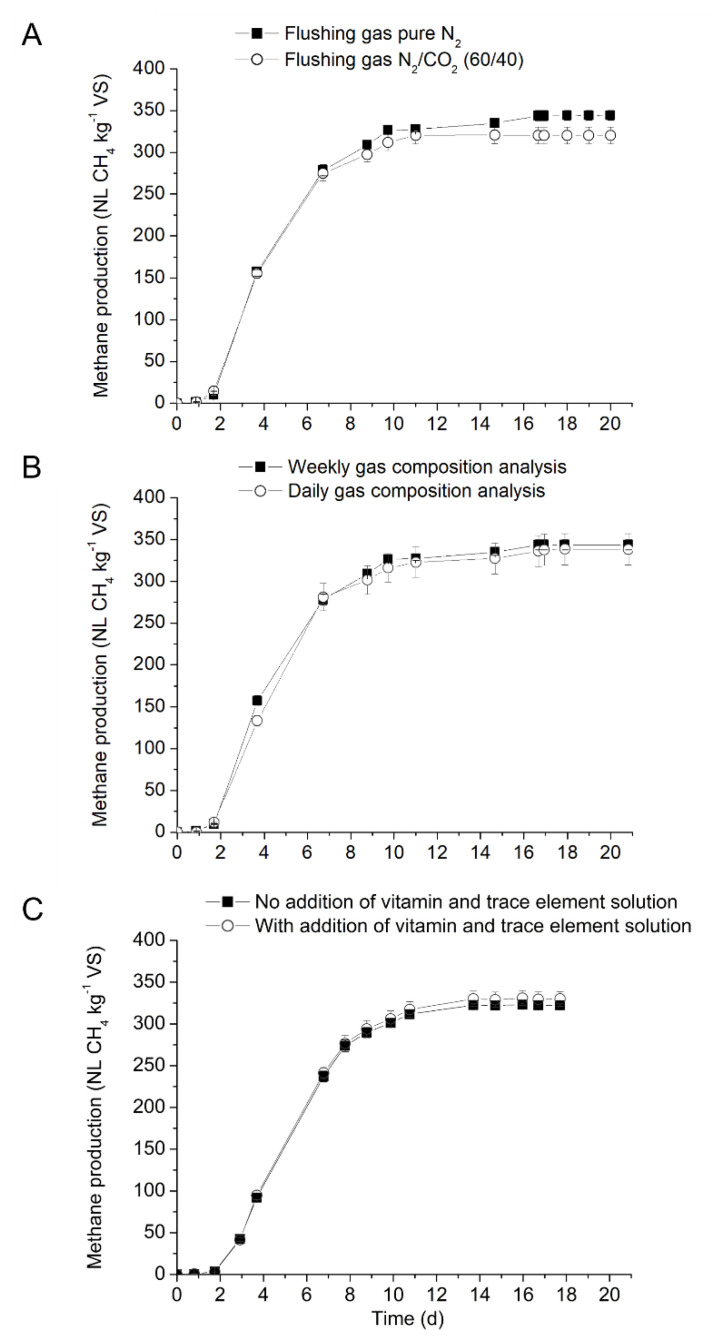
Influence operational factors on the mesophilic BMP of cellulose performed with Inoculum 1, (**A**) flushing gases, (**B**) analysis frequency, (**C**) vitamin and trace element.

The effect of several operational factors on the methane potential (NL CH_4_ kg^−1^ VS) of cellulose is presented in Table 3 and Figure 1A–C.

At first, the effect of two flushing gas on BMP from cellulose was investigated with pure N_2_ and a mixture of N_2_ and CO_2_ (60/40 *v*/*v*). Methane production from blank and cellulose started after a lag phase of two days and maximum methane production was reached after 17 days of incubation (Figure 1A). Methane yields from assays flushed with pure N_2_ was 7% higher than from assays flushed with gas mixture of N_2_ and CO_2_. Mean methane yields obtained for cellulose with N_2_/CO_2_ and N_2_ flushing gases were 321 and 344 NL CH_4_ kg^−1^ VS, respectively and significantly different (*p*.value = 0.020 < 0.05). The results are in contradiction to the results reported by Koch et al. [14,15]. In the above study, methane production from blank assays flushed with N_2_/CO_2_ or pure CO_2_ was higher than with assays flushed with pure N_2_ [13,14]. Indeed, Koch et al. (2016) highlighted that the CH_4_ yields increased linearly with increase in the CO_2_ concentration in the flush gas reaching a 30% higher yield at pure CO_2_ relative to pure N_2_ headspace conditions. Similarly, Koch et al. (2015) have also investigated the effect of three modalities: flushing with N_2_ gas, a mixture of N_2_ and CO_2_ (80/20 *v*/*v*), and no flushing. It was concluded that the removing of oxygen in the headspace is crucial to avoid aerobic respiration and the presence of CO_2_ in the flushing gas significantly increased the methane production by over 20% in comparison to the flushing with pure N_2_. However, no significant results on the effect of flushing gas was reported by other authors or in inter-laboratory studies [5,15,32]. In our study, flushing the headspace with pure N_2_ had shown better results and will be used for further tests.

The results on the frequency of biogas analysis are presented in Figure 1B and Table 3. For cellulose control, the quantity and the composition of the biogas produced was determined and the results were compared to the weekly measurement. As can be observed, no significant difference between the different frequencies of biogas measurement was noticed (*p*.value 0.522 > 0.05). Higher standard deviation was noticed when the biogas composition was analyzed every day, reflecting the multiple small measurement bias (even if an automatic injection of gas).

The effect of trace elements and vitamins in the anaerobic medium was investigated and the results are presented in Figure 1C and Table 3. Results showed that the inoculum in the present study has sufficient trace elements and vitamin due to its origin. Previous studies showed that some inoculum were deficit in trace elements and vitamins, and therefore supplementation of trace element and vitamins was required to produce satisfactory biogas production [9]. Macro- and micronutrients are necessary for cell growth, especially methanogens, which require special micronutrients for optimal growth and reproduction [33]. To evaluate the importance of trace elements and vitamins, BMP study was performed with APESA inoculum. The results of the biogas production from APSEA inoculum with and without supplementation of vitamin and trace elements are presented in Figure 1C. Results showed that the effect of vitamin and trace elements supplementation had no significant effect (*p*.value 0.234 > 0.05) on the kinetics or final methane production. Methane yields of 323 and 331 NL CH_4_ kg^−1^ VS were obtained from assays without or with vitamins and trace elements supplementation, respectively. These results confirm that the APESA inoculum used in this study had sufficient quantities of vitamins and trace elements and therefore do not need supplementation. Parra-Orobio et al. (2018) have investigated the effect of trace elements supplementation on anaerobic digestion of food waste using three different inocula (granular sludge from UASB reactor treating sugar industry wastewater, granular sludge from UASB reactor treating slaughterhouse wastewater, and flocculent sludge from USAB treating municipal wastewater) [34]. Results in the above study showed that addition of trace elements did not have any significant difference in methane yields with inoculum sourced from USAB reactors treating sugar industry but improved methane production from the same reactor treating slaughter of cattle and pigs [34]. Nonetheless, in most cases, if no preliminary analysis is done, it is unclear whether BMP inoculum or substrates tested will have sufficient nutrients available and supplementation can be recommended at the start of the BMP assays [9].

### 3.2. Influence of the Source of Inoculum on Methane Production 

**Figure 2:**
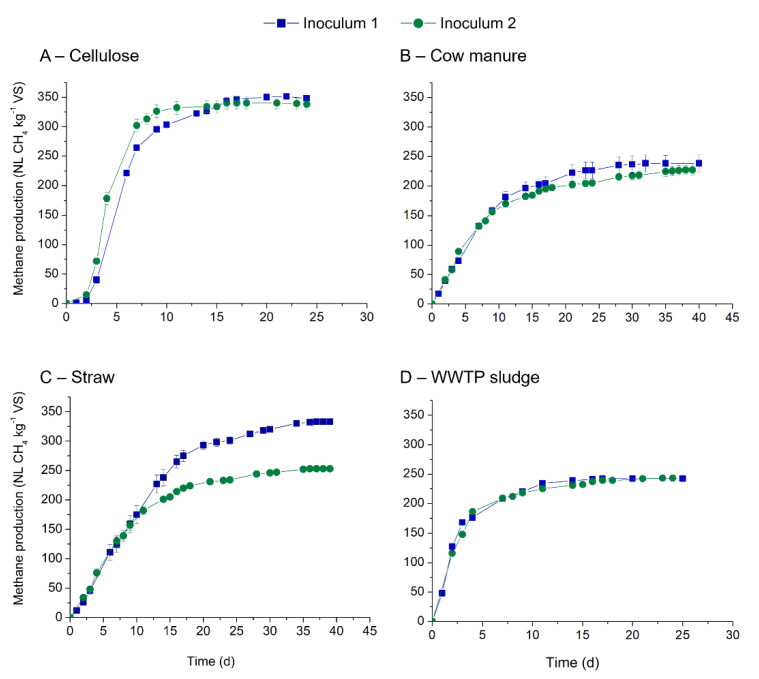
Methane production obtained for two inoculum using four substrates: (**A**) cellulose, (**B**) cow manure, (**C**) straw and (**D**) WWTP sludge.

The source of inoculum influences the chemical and microbial composition and therefore affect the kinetics and methane production during the BMP testing. In our study, the effect of two inocula on the kinetics and methane production from cellulose, cow manure, straw and WWTP sludge was studied, and the results are presented in Figure 2 and Table 4. The kinetic constants as well as the theoretical BMP for each substrate are presented in Figure 2 and Table 4.

With both inocula, methane production started immediately from all assays except for cellulose. Mean maximum methane production obtained for the studied substrates ranged from 227 to 348 NL CH_4_ kg^−1^ VS (Table 4). Cellulose was shown to be well degraded by the two tested inocula reflecting the presence of diversified microflora and high activity of the initial microbial community. For cow manure, methane production reached 227 and 238 NL CH_4_ kg^−1^ VS with similar rates of kinetics. Similar results were also noticed with WWTP sludge, with methane yields ranging from 242 to 243 NL CH_4_ kg^−1^ VS. Only wheat straw showed a clear significant difference in methane yields between the two inoculum sources (*p*.value 0.0008 < 0.05). Inoculum 1 resulted in higher methane (333 NL CH_4_ kg^−1^ VS) compared with the inoculum 2 (253 NL CH_4_ kg^−1^ VS). However, the kinetics of methane production were more or less the same (Table 4). With other substrates, no significant difference in methane yields was noticed as all the *p*.values were above 5% (*p*.values were 0.109, 0.656, 0.165 for cellulose, cow manure and WWTP sludge, respectively). 

Methane yields obtained in the present study are similar to the yields of 129 to 366 NL CH_4_ kg^−1^ VS reported in the literature for cow manure [25,29,35]. The composition of cow manure is dependent on the animal species, the bedding material used and the feed composition. For example, methane yields from animal bedding alone can range from 244 to 416 NL CH_4_ kg^−1^ VS depending on the inoculum and their source used [36]. Nonetheless, two publications found similar mean for large set of manure sample. Mortreuil et al. (2017) measured the methane performance of 58 solid manures with an ISR of 2.8 and found a mean of 225 NL CH_4_/kg VS (range of 129–366 NL CH_4_/kg VS) [35]. Similarly, Rodrigues et al. (2018) performed BMP tests on 12 manures (ISR at 2) and obtained a mean of 211 NL CH_4_/kg VS (from 154 to 325 NL CH_4_/kg VS) [37]. These two results are clearly similar to the ones obtained here. Similarly, for straw, methane yields ranging between 177 and 301 NL CH_4_ kg^−1^ VS were reported in the literature and were shown to be dependent on the ISR used and the inoculum sources. Moreover, straw composition is dependent on the crop variety and the harvesting time. For instance, methane yields can vary from 219 to 334 NL CH_4_ kg^−1^ VS for maize or 246 to 274 NL CH_4_ kg^−1^ VS for ensiled sorghum for four different inocula [25,31]. In an inter-laboratory study, wheat straw incubated with different inocula at a fixed ISR of 1, reported methane yields of 280–303 NL CH_4_ kg^−1^ VS [38]. As for WWTP, a marge range of methane production are available in the literature: from 13 to 711 NL CH_4_/kg VS [37,39]. If we focus on means obtained for sets of more or equal to 10 different samples, methane production found where 172 NL CH_4_/kg VS (30 samples) [39], 353 NL CH_4_/kg VS (19 samples) [40], 411 NL CH_4_/kg VS (10 samples) [37] and 181 NL CH_4_/kg VS (20 samples) [41]. This variation in methane yields obtained is due to differences in composition of WWTP sludge and the treatment used (flocculation, primary or secondary digester, retention time, etc.). Our results are in the range of the means obtained.

The influence of source of inoculum on methane yields from different substrates reported in the literature, a resume is presented in Supplementary Data. In the present study, except for straw sample, source of inoculum had no significant difference in methane yields for the studied substrates. Indeed, for straw, methane potential of 333 and 253 NL CH_4_/kg VS were respectively observed for inoculum 1 and 2. Such difference can be certainly explained by the differences of inoculum origin, the composition, the microbial community structure, and microbial activity [33].

Differences link to inocula were seen in the inter-laboratory studies [4,11,38]. For instance, Moset et al. [25] had tested the influence of four different inocula on different substrates (cellulose, cow manure, maize silage, and wheat straw). Inocula tested were fed with different feedstock, one with only sludge and the three others in co-digestion with manure, maize/grass and/or industrial waste. The author reported that raw inoculum source had a significant impact on both yields and kinetics parameters on wheat straw (177 to 280 NL CH_4_ kg VS^−1^ with ISR ranging to 0.91 at 1.21). In contrast, Hülsemann et al. [20] tested five different sources of inoculum with five substrates and reported significant different results for dried maize silage, triglyceride fodder, concentrated folder, and cellulose on methane yields while the inoculum source had no impact on methane yields from hay [20]. Parra-Orobio et al. [33] studied the effect of three different inoculum sources on the methane yields of food wastes. Results showed that use of an inoculum from a sugar mill WWTP (Inoculum I) and inoculum obtained from a treatment plant that treats wastewater from cattle slaughter (Inoculum II) had similar methane yields of 144 and 149 NL CH_4_ kg VS^−1^ whereas the inoculum from a municipal WWTP (Inoculum III) had the lowest methane yields 100 NL CH_4_ kg VS^−1^ suggesting that the source of inoculum can affect the methane yields [34]. In a similar study, De Vrieze et al. [9] studied the effect of four different inocula collected from full-scale biogas plants and their influence on the methane yields of four substrates (molasses, bio-refinery waste, liquid manure, and high-rate activated sludge). Results showed that the inoculum source had no significant effect on the methane yields of molasses and bio-refinery waste but showed significant difference in methane yields with liquid manure and activated sludge (i.e., methane production ranged from 304 to 455 NL CH_4_ kg VS^−1^ for sludge). These results suggest that the influence of inoculum source on methane yields is dependent on several factors, such as microbial community and, enzymatic activities within the inoculum, and more specifically of the methanogenic ones [19] and the concentration of trace elements and vitamins in the inoculum [9]. On the contrary, Sambusiti et al. [36] investigated the effect of four different sources of inoculum (urban, agricultural, mixture of agricultural and urban, granular) on the methane potential of ensiled sorghum and reported than methane yields were not influenced by the tested inocula while methane production rate was affected by the source of inoculum [42]. Thus, the results on the effect of source of inoculum on methane yields are contradictory and further research is needed to better understand the influence of inoculum source, as methane yields are also dependent on the initial biodegradability of the substrate.

### 3.3. Influence of Freezing for Feedstock Conservation

**Figure 3:**
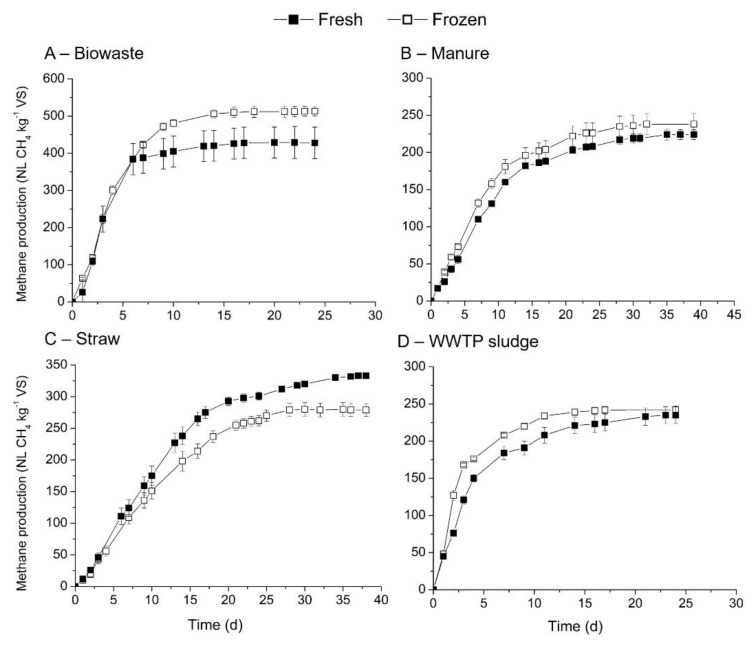
Comparison of methane production and kinetics between fresh and freezing/thawing cycle substrates: (**A**) biowastes, (**B**) cow manure, (**C**) straw and (**D**) WWTP sludge.

Freezing is a usual practice to conserve a feedstock when it cannot be used within a few days after reception [12]. The freezing process can damage the structure of the substrate as the formation of water crystals occurs [26]. However, little information is available regarding the influence of this conservation mode on methane production. The effect of freezing/thawing cycle on the methane yields of biowaste, cow manure, straw and WWTP substrates was studied and compared with fresh substrates. Methane production and kinetic constants are indicated in Table 5 and Figure 3. These substrates were selected as they had different solids content (Table 2).

Mixed results were noticed on the influence of freezing/thawing cycle on methane production rates and yields. For instance, methane yields increased for cow manure (6.25%), WWTP sludge (2.98%) and biowaste (19.58%) but decreased for straw (18.9%). Student test results showed *p*.value at 0.016 < 0.05 for straw. However, the kinetics of methane production was shown to be dependent on the substrate composition (Table 5). Biowaste had similar kinetics between the fresh (0.311 d^−1^) and freezing/thawing materials (0.314 d^−1^). On the other hand, the freezing/thawing cycle improved the kinetics of methane production rates (Table 5). One of the possible explanations is an increase of organic matter accessibility for the microorganisms due to ice formation leading to porosity improvement.

First, as methane potentials of three substrates have been compared to literature data, only biowaste will be discussed in this section. A large set of data regrouping biowaste in general was performed in the literature, on freeze samples mostly with an ISR ranging from 2 to 5 (mainly at 2) [35,39,40,43]. Methane production evaluated ranged from 96 to 900 NL CH_4_/kg VS showing the high variability of this type of substrates. Again, focusing on means, 329 NL CH_4_/kg VS (for 10 samples) [43], 338 NL CH_4_/kg VS (for 25 samples) [35], 370 NL CH_4_/kg VS (for 20 biowastes) [39] and 461 NL CH_4_/kg VS (for 50 biowastes) [40] have been obtained. We obtained a higher value of mean indicated, 513 NL CH_4_/kg VS, which is within the wide range of this substrate.

The effect of freezing/thawing cycle for different substrates showed an improvement or a reduction of the methane production (summarized in Supplementary Data). Only three of the four substrates can be compared to the literature, as no information is available for frozen cow manure. For WWTP sludge, freezing had a significant positive impact on methane production (+46% [28]). This difference can be explained by the different freezing/thawing process (−15 °C and 6 °C) used in the present study compared with conditions of −25 °C and 20 °C used in [28] or by the initial sludge characteristics (difference in TS concentration). With respect to straw, a decrease in methane yields 8% (non-significant) after freezing/thawing, as reported in the literature [25]. For biowaste, an improvement in the methane production was reported in the literature: +20% (this study), +4% [12] and +7% [26]; but not always significant. Thus, the influence of the freezing/thawing cycle on the kinetics and methane yields may be linked to the composition and the water content of the substrate.

## 4. Conclusions

In this study, the influence of BMP operational parameters, source of inoculum, and feedstock conservation methods on kinetics of methane production rates and methane yields was studied. Results showed that the type of gas used for flushing headspace of BMP assays with pure N_2_ or a mixture N_2_/CO_2_ had showed a significant difference on the methane yields of the studied substrates. On the other hand, other operational parameters such as gas analysis frequency, trace elements, and vitamin supplementation showed no significant influence on the kinetics and methane yields. Although the source of inoculum did not affect the methane potential of three studied substrates (cellulose, cow manure and biowaste), a significant difference on methane production from straw was noticed. Finally, the effect of feedstock conservation by freezing/thawing cycle showed contrasting results on the methane yields for the four substrates suggesting that concentration of water in substrate can have an effect on the methane yields from frozen/thawed substrates. This study thus confirms the necessity to work on the development and implementation of inter-laboratory BMP testing protocol.

## Figures and Tables

**Table 1:** Characterization of the two inoculum in terms of their main physico-chemical properties.

Parameters	Units	Inoculum 1	Inoculum 2
Temperature	°C	38	38
pH		7.9	7.5
Redox	mV ENH^−1^	−433	−382
TS	% weight	3.6 ± 0.01	4.4 ± 0.01
VS	% TS	64.6 ± 0.7	71.7 ± 0.1
Alkalinity	g CaCO_3_ L^−1^	5.7	5.3
N-NH_4_^+^	g L^−1^	1.5	1.3
VFAs	g L^−1^	0.1	2.4

**Table 2:** Substrate composition and Theoretical Methane Potential (TMC). CHNS standard deviation were inferior of 0.2%.

Substrates	TS (% w)	VS (% TS)	C (%)	H (%)	N (%)	S (%)	TMC (NL CH_4_ kg^−1^ VS)
Biowastes	11.3 ± 1.3	89.9 ± 1.3	47.7	7.9	4.6	0.0	593
Cellulose	87.1 ± 0.1	100.0 ± 0.1	42.6	6.1	0.0	0.0	388
Cow manure	25.5 ± 0.01	78.2 ± 0.01	41.1	5.8	2.0	0.0	536
Straw	91.0 ± 0.1	91.0 ± 0.5	43.2	6.0	0.6	0.0	467
WWTP sludge	20.1 ± 0.01	78.2 ± 0.01	39.9	6.2	6.9	0.0	532

**Table 3:** Impact of the operational factor on the BMP kinetic and methane production of cellulose.

Operational Factors	Condition	Methane Production (NL CH_4_ kg^−1^ VS)	Kinetic Rate (d^−1^)
Flushing gas	Pure N_2_	344 ± 6	0.324 ± 0.006
	N_2_/CO_2_	321 ± 10	0.371 ± 0.012
Gas analysis	Weekly	344 ± 6	0.352 ± 0.006
	Daily	338 ± 19	0.397 ± 0.022
Vitamin and trace element solution	No addition	323 ± 3	0.370 ± 0.003
	With addition	331 ± 9	0.355 ± 0.010

**Table 4:** Effect of source of inoculum on the kinetics and methane production.

	Inoculum 1		Inoculum 2	
Substrates	Methane Production (NL CH_4_ kg^−1^ VS)	Kinetic Rate (d^−1^)	Methane Production (NL CH_4_ kg^−1^ VS)	Kinetic Rate (d^−1^)
Cellulose	348 ± 2	0.209 ± 0.007	340 ± 10	0.452 ± 0.016
Cow manure	238 ± 14	0.128 ± 0.001	227 ± 8	0.106 ± 0.004
Straw	333 ± 4	0.112 ± 0.002	253 ± 3	0.115 ± 0.001
WWTP sludge	242 ± 5	0.377 ± 0.002	243 ± 3	0.339 ± 0.007

**Table 5:** Influence of the freezing/thawing process on biogas production and kinetics.

	Fresh		Freezing/Thawing Cycle	
Substrates	Methane Production (NL CH_4_ kg^−^^1^ VS)	Kinetic Rate (d^−^^1^)	Methane Production (NL CH_4_ kg^−^^1^ VS)	Kinetic Rate (d^−^^1^)
Biowastes	429 ± 42	0.311 ± 0.006	513 ± 13	0.314 ± 0.031
Cow manure	224 ± 7	0.117 ± 0.006	238 ± 14	0.136 ± 0.006
Straw	333 ± 4	0.102 ± 0.004	280 ± 11	0.113 ± 0.012
WWTP sludge	235 ± 11	0.203 ± 0.011	242 ± 5	0.329 ± 0.008

## Data Availability

Data is contained within the article or Supplementary Material.

## Supplementary Materials

The following are available online at https://www.mdpi.com/article/10.3390/bioengineering8110176/s1, Table S1. The influence of source of inoculum on methane production from different substrates reported in the literature. % referred to wet weight. n.i for not indicated. Table S2. The effect of freezing/thawing cycle on methane production of different substrates. Where n.i: not indicated; Δ is calculated by (V_CH4_ frozen − V_CH4_ fresh)/V_CH4_ fresh; +: V_CH4_ frozen > V_CH4_ fresh; −: V_CH4_ fresh > V_CH4_ frozen and *: significant impact.

## Abbreviations

BMP	Biochemical Methane Potential
ISR	Inoculum Substrate Ratio
TS	Total Solid
VS	Volatile Solid
WWTP	Wastewater Treatment Plant
