# Supplementary material for: Impact of Operational Factors, Inoculum Origin, and Feedstock Preservation on the Biochemical Methane Potential
Source: Bioengineering (Basel). 2021 Nov 5;8(11):176. doi: 10.3390/bioengineering8110176 (PMC8614716; doi:10.3390/bioengineering8110176)
Supplement: Supplementary file 1 [file bioengineering-08-00176-s001.zip › bioengineering-1419264-supplementary.pdf]

Impact of Operational Factors, Inoculum Origin, and Feedstock Preservation on the Biochemical Methane Potential

Audrey Lallement, Aline Siaud, Christine Peyrelasse, Prasad Kaparaju, Blandine Schraauwers, Samuel Maunas and Florian Monlau

Table S1. The influence of source of inoculum on methane production from different substrates reported in literature. % referred to wet weight. n.i for not indicated.

**Inoculum sources**	**BMP test conditions or pilots**	**Methane production (NL CH_4 kg^{-1} VS)**				**Ref.**
Substrates			Liquide pig manure	Sludge		
**Industrial digester** (brewery waste)	54°C, ISR = 2		330 ± 38	406 ± 45		[1]
**Agricultural digester** (manure)			137 ± 28	304 ± 45		
**Territorial digester**			n.i	347 ± 14		
**Agricultural digester** (manure and energy crop)			n.i	455 ± 26		
Substrates		Cellulose	Cow manure	Maize silage	Wheat straw	
**Horsens** (100% Sludge municipal WWTP)	35°C, ISR = 0.91	328	234	299	280	[2]
**Bånlev** (75% animal manure and 25% industrial waste)	35°C, ISR = 0.79	328	136	219	177	
**Foulum** (80% manure and 20% maize and grass)	53°C, ISR = 1.21	282	245	334	301	
**Thorsø** (75% animal manure, 23% industrial waste and 2% maize/grass)	53°C, ISR = 0.93	399	249	287	266	
Substrates			Ensiled sorghum			
**Waste activated sludge digester (WW)**	35°C, ISR = 1		248 ± 20			[3]
**Agricultural biogas plant (AGR)**			274 ± 18			
**UASB reactor treating wastewater (GR)**			246 ± 15			
**A mix of WW and AGR**			265 ± 12			
Substrates			Cow manure			
**From Foulum research center**	Pilot thermophilic, ISR = 1		325 ± 17			[4]
	Pilot mesophilic, ISR = 1		325 ± 22			
Substrates	Slurries from	dairy cow	fattening pig		forrowing sow	
**Sludge municipal WWTP**	30°C, ISR = 0.5 - 1	257	261		416	[5]
**Agricultural : Swine slurry**		244	272		408	

Table S2. The effect of freezing/thawing cycle on methane production of different substrates. Where n.i: not indicated; Δ is calculated by (V_{CH4} frozen – V_{CH4} fresh)/ V_{CH4} fresh; +: V_{CH4} frozen > V_{CH4} fresh; -: V_{CH4} fresh > V_{CH4} frozen and *: significant impact.

Substrates	TS	Freezing conditions	BMP test condition or pilots	Methane potential (NL CH_4 kg^{-1} VS)			Ref.
	% Weight			Fresh	Frozen	Δ	
**Green waste grinded at 4 mm**	n.i	n.i	Mesophilic ISR = 4	107	113	+6%$^{+}$	[6]
**Green waste grinded at 10 mm**	n.i			117	120	+3%$^{+}$	
**Food waste from restaurant and supermarket (lavures)**	n.i			536	555	+4%$^{+}$	
**Food waste**	17%	24h at -20°C then 12h at 25°C	Pilot mesophilic	300	320	+7%$^{+*}$	[7]
**Grass**	17%	6 days at -18°C	Thermophilic ISR = 1	368	397	+8%$^{+}$	[2]
**Maize**	29%			431	363	-16%$^{-*}$	
**Wet straw**	86%			346	317	-8%$^{-}$	
**WWTP sludge**	3%	24h at -25°C then 12h at 20°C	Pilot mesophilic	497	727	+46%$^{+*}$	[8]

REFERENCE

1. de Vrieze, J.; Raport, L.; Willems, B.; Verbrugge, S.; Volcke, E.; Meers, E.; Angenent, L.T.; Boon, N. Inoculum selection influences the biochemical methane potential of agro-industrial substrates. *Microb. Biotechnol.* **2015**, *8*, 776–786.

2. Moset, V.; Al-zohairi, N.; Møller, H.B. The impact of inoculum source, inoculum to substrate ratio and sample preservation on methane potential from different substrates. *Biomass Bioenergy* **2015**, *83*, 474–482.

3. Anderson, G.K.; Yang, G. Determination of bicarbonate and total volatile acid concentration in anaerobic digesters using a simple titration. *Water Environ. Res.* **1992**, *64*, 53–59.

4. Achinas, S.; Euverink, G.J.W. Theoretical analysis of biogas potential prediction from agricultural waste. *Resour. -Effic. Technol.* **2016**, *2*, 143–147.

5. Vedrenne, F.; Béline, F.; Dabert, P.; Bernet, N. The effect of incubation conditions on the laboratory measurement of the methane producing capacity of livestock wastes. *Bioresour. Technol.* **2008**, *99*, 146–155.

6. Hack, G.; de Fruteau Laclos, H.; Holliger, C. *Détermination de la Production de Méthane d'installations de Digestion Industrielles à Partir de Tests BMP en Laboratoire*; Office Fédérale de l'Energie: Ittigen, Switzerland, 2015, 1–76.

7. Stabnikova, O.; Liu, X.Y.; Wang, J.Y. Digestion of frozen/thawed food waste in the hybrid anaerobic solid–liquid system. *Waste Manag.* **2008**, *28*, 1654–1659.

8. Montusiewicz, A.; Lebiocka, M.; Rożej, A.; Zacharska, E.; Pawłowski, L. Freezing/thawing effects on anaerobic digestion of mixed sewage sludge. *Bioresour. Technol.* **2010**, *101*, 3466–3473.
